# Supplementary material for: Real-time self-supervised denoising for high-speed fluorescence neural imaging
Source: Nat Commun. 2025 Oct 24;16:9396. doi: 10.1038/s41467-025-64681-8 (PMC12552699; doi:10.1038/s41467-025-64681-8)
Supplement: Supplementary file 1 — Supplementary Information [file 41467_2025_64681_MOESM1_ESM.pdf]

Supplementary Information

Real-time self-supervised denoising for high-speed neural fluorescence imaging

Yiqun Wang[1], Yuanjie Gu[1], Jianping Wang[1], Ang Xuan[1], Cihang Kong[2], Weiqun Fang[3], Dongyu Li[4], Dan Zhu[5], Fengfei Ding[6], and Biqin Dong[1]

[1]The Academy for Engineering and Technology, Yiwu Research Institute, Fudan University, Shanghai, China
[2]Institute for Translational Brain Research, Fudan University, Shanghai, China
[3]College of Basic Medical Sciences, Shanghai Jiao Tong University, Shanghai, China
[4]School of Optical Electronic Information, Advanced Biomedical Imaging Facility, Huazhong University of Science and Technology, Wuhan, Hubei, China
[5]Britton Chance Center for Biomedical Photonics, MoE Key Laboratory for Biomedical Photonics, Wuhan National Laboratory for Optoelectronics, Advanced Biomedical Imaging Facility, Huazhong University of Science and Technology, Wuhan, Hubei, China
[6]Department of Pharmacology, Shanghai Medical College, Fudan University, Shanghai, China

Supplementary Section 1: Evaluating the performance of different SNR and imaging speeds.

Supplementary Section 2: Evaluation of hyperparameters.

Supplementary Section 3: Synthetic calcium and voltage imaging data generation.

Supplementary Section 4: Quantitative comparison of different network variants based on ablation experiments.

Supplementary Section 5: Evaluating the sampling strategy of FAST.

Supplementary Section 6: Evaluating the network architecture of FAST.

Supplementary Section 7: Evaluating the data dependency of FAST.

Supplementary Section 8: Real-time denoising of two-photon calcium imaging in mice with FAST.

Supplementary Section 9: The denoising performance of different methods on calcium imaging data.

Supplementary Section 10: FAST enhances single-cell voltage imaging, with a comparison to electrophysiological signals.

Supplementary Section 11: FAST enhances population voltage imaging of the zebrafish larval spinal cord.

Supplementary Section 12: FAST enhances two-photo dual-channel volumetric imaging of astrocytes.

Supplementary Section 13: Evaluating the impact of motion artifacts on FAST denoising performance.

Supplementary Figure 1: Evaluating the performance of different SNR and imaging speeds.

Supplementary Figure 2: Evaluation of hyperparameters.

Supplementary Figure 3: Network architecture of FAST.

Supplementary Figure 4: Quantitative comparison of different network variants based on ablation experiments.

Supplementary Figure 5: In silico simulation of calcium imaging data.

Supplementary Figure 6: Evaluating the sampling strategy of FAST.

Supplementary Figure 7: Evaluating the network architecture of FAST.

Supplementary Figure 8: Evaluating the data dependency of FAST.

Supplementary Figure 9: FAST graphical user interface overview.

Supplementary Figure 10: Evaluation of FAST performance in fast dynamics.

Supplementary Figure 11: Evaluating the impact of motion artifacts on FAST denoising performance.

Supplementary Table 1: List of datasets analyzed in this study.

Supplementary Table 2: List of denoising methods compared in this study.

Supplementary Video 1: Real-time denoising of two-photon calcium imaging in mice with FAST.

Supplementary Video 2: The denoising performance of different methods on calcium imaging data.

Supplementary Video 3: FAST enhances single-cell voltage imaging, with a comparison to electrophysiological signals.

Supplementary Video 4: FAST enhances population voltage imaging of the zebrafish larval spinal cord.

Supplementary Video 5: FAST enhances two-photo dual-channel volumetric imaging of astrocytes.

Supplementary Section 1: Evaluating the performance of different SNR and imaging speeds.

For the comparison experiments, we used simulated calcium and voltage imaging data under varying SNR conditions. The sampling rates for calcium imaging were set at 1, 30, and 60 Hz. Specifically, 60 Hz calcium imaging data were first simulated, followed by extraction of 1Hz and 30 Hz data through frame subsampling. Similarly, for voltage imaging, data were generated at sampling rates of 100, 500, and 1,000 Hz. Initially, 1,000 Hz voltage imaging data were simulated, and 100 Hz and 500 Hz data were subsequently extracted through frame subsampling.

The performance of FAST remains stable across different speeds, suggesting that it is well-suited for applications that require flexibility in imaging rates. The ability to handle low-SNR conditions at 1Hz highlights FAST's strength in reducing noise even under challenging conditions. FAST effectively denoises voltage imaging data at very high speeds (1,000 Hz), making it suitable for experiments that require real-time or near real-time imaging. These findings highlight the robustness of FAST as a denoising algorithm for both calcium and voltage imaging, making it a versatile tool for use across a wide range of experimental conditions.

Supplementary Section 2: Evaluation of hyperparameters.

To quantitatively assess the impact of trade-off parameters in the model, we performed simulations using various hyperparameter configurations. Specifically, we tested four different sliding window widths (C values): 2, 4, 8, and 16. For each C value, we evaluated all possible sliding window stride sizes (S values). The performance of these configurations was measured using three metrics: PSNR, SSIM, and FPS. This allowed us to compare the denoising quality and processing speed under different parameter settings.

With the increase in sliding window width (C), PSNR generally improves, indicating that larger window sizes lead to better denoising quality. A similar trend is observed in SSIM, where larger C values result in higher structural similarity between the denoised image and the real image. However, beyond $C = 8$, the improvement in SSIM initially increases but then decreases as the sliding window stride size (S) continues to grow. This suggests that while a larger window size enhances the denoising performance, the interaction with the stride size can introduce diminishing returns or even performance degradation in structural similarity. There is a clear trade-off between processing speed and denoising quality: smaller C values lead to slower FPS, particularly when smaller stride sizes (S) are used, where computational costs rise sharply. Overall, the data suggest that careful selection of hyperparameters is necessary to strike a balance between optimizing denoising performance and maintaining real-time processing speeds.

Supplementary Section 3: Synthetic calcium and voltage imaging data generation.

Simulated calcium and voltage imaging data were generated by first creating clean videos (ground truth) and then adding Poisson-Gaussian noise. For realistic spatial neuron contours akin to those in the mouse brain, we employed the NAOMI[1] simulator, which was initially developed for simulating two-photon calcium imaging datasets.

For simulating calcium imaging, we set the volume size to 500 × 500 × 50 μm^3 and the imaging depth to 200 μm, placing the imaging plane at the middle of the simulated volume. The point spread function (PSF) parameters were defined with an objective numerical aperture (NA) of 0.8

and a PSF NA of 0.6. The two-photon microscopy power was set to 50 mW. Temporal dynamics were controlled by setting the number of time steps to 6000 and the frame rate period to 1/60 seconds, representing a sampling frame rate of 60 Hz. The average firing rate was modulated to 0.6, and motion artifacts were disabled to maintain focus on the calcium signal simulation. Other parameters were kept at their default values.

For simulating voltage imaging, we adapted the code to generate voltage transients instead of calcium transients as the temporal component. The volume size was adjusted to 300 × 300 × 50 μm^3, with the same imaging depth of 200 μm. We maintained the PSF parameters with an objective NA of 0.8 and a PSF NA of 0.6. The two-photon microscopy power remained at 50 mW. The temporal settings were modified to match the characteristics of voltage imaging, with the number of time steps kept at 6000 but the frame rate period set to 1/1,000 seconds, achieving a sampling rate of 1,000 Hz. The average firing rate was reduced to 0.25 to reflect the distinct nature of voltage signal dynamics. As with calcium imaging, motion simulation was turned off.

By adding Poisson-Gaussian noise of varying intensities to these simulations, we generated noisy videos with different signal-to-noise ratios (SNRs).

Supplementary Section 4: Quantitative comparison of different network variants based on ablation experiments.

To systematically assess the impact of key architectural choices, we conducted a series of ablation experiments, as summarized in Figure X. Each variant represents a modification to one aspect of the network design, and the results are visualized in terms of denoising performance (SNR), model size, and inference speed.

The data used for these ablation studies were generated using the NAOMI simulator to produce noise-free calcium imaging sequences. As stated in Supplementary Section 3, to simulate realistic imaging conditions, we subsequently added synthetic Poisson-Gaussian noise to the data, ensuring a controlled and reproducible evaluation environment.

The FAST model (our baseline, highlighted in red) achieves a strong balance among all three metrics: it delivers a substantial improvement in SNR (23.27 dB) over the raw input data (11.11 dB, indicated by the dashed line), while maintaining an exceptionally compact parameter count (0.0136M) and fast inference. In contrast, replacing group convolutions with standard convolutions dramatically increases the parameter count (over 25-fold) and reduces SNR, underscoring the effectiveness of group convolutions for both efficiency and performance.

Adding a top-level skip connection yields a marginal SNR gain but nearly doubles the model size, suggesting diminishing returns relative to increased complexity. Varying the number of down-sampling steps reveals that reducing depth to two severely compromises performance, while increasing depth to four offers only slight SNR improvement at the cost of a larger model. Similarly, increasing channel width to 128 improves SNR but more than doubles the parameter count, whereas reducing it to 32 channels leads to performance degradation.

Overall, these results demonstrate that the FAST architecture is well-calibrated for the specific demands of biological fluorescence microscopy denoising: it achieves competitive performance with minimal computational and memory requirements. The ablation studies validate our design choices, confirming that further increases in model complexity do not yield proportional benefits for this application scenario.

Supplementary Section 5: Evaluating the sampling strategy of FAST.

For the comparison experiments, we utilized simulated calcium imaging data at 30 Hz with an input image SNR of -1.25 dB. Each model was evaluated using the same network architecture, trained for 100 epochs, with the only variation being the sampling strategies employed. In temporal sampling, input and target are adjacent frames in time. In spatial sampling, input and target are adjacent sub-frames extracted from the same frame. The FAST sampling strategy utilizes the spatiotemporal sampling strategy proposed in this study.

The FAST sampling strategy integrates both temporal and spatial information, making it more effective than either temporal or spatial sampling alone. This leads to improved denoising performance, especially in dynamic imaging applications like calcium imaging. Since all models use the same network architecture, the results highlight that the sampling strategy plays a crucial role in the quality of the denoising outcome. The superior performance of the FAST strategy underscores the importance of designing sampling methods that capture both spatial and temporal dimensions of the data.

Supplementary Section 6: Evaluating the network architecture of FAST.

For the comparison experiments, we utilized simulated calcium imaging data at 30 Hz with an input image SNR of -1.25 dB. Both the classical U-Net network and the FAST network proposed in this paper were trained for 100 epochs. While both the U-Net and FAST networks can capture spatiotemporal information through the same sampling method, the FAST network's architecture is better at processing this information to achieve superior denoising results. The architectural advantages of FAST are clear, as it outperforms U-Net even when both networks operate under identical conditions. This demonstrates that the FAST network's design is better suited for tasks where spatiotemporal resolution is critical, such as calcium imaging.

Supplementary Section 7: Evaluating the data dependency of FAST.

For the comparison experiments, we utilized simulated calcium imaging data at 30 Hz with an input image SNR of -1.25 dB. Each model was trained for 100 epochs and the last epoch was used for comparison. FAST's performance improves with increasing amounts of training data (frames), but beyond a certain point, the gains become marginal. This suggests that while more data is generally beneficial, there is an optimal window where the trade-off between computational cost and performance gain should be considered. Overall, these results show that FAST can effectively utilize time-lapse imaging data to enhance denoising performance, making it suitable for a variety of sample sizes.

Supplementary Section 8: Real-time denoising of two-photon calcium imaging in mice with FAST.

We used Matlab-based ScanImage software for image acquisition. Before starting the experiment, imaging control parameters were preset. FAST was then launched, and the data read and save paths, model path, and buffer size were configured. The FAST system consists of two main components: a Matlab-based application and a Python-based deep learning denoising module. Acquired image data is buffered and accessed in real time by the Python module for denoising and visualization. During the experiment, both the raw and denoised images are displayed simultaneously, enabling real-time comparison. After processing, the denoised data is

automatically saved to disk. The video demonstrates an acquisition rate of 30 Hz at 512×512 pixels and is shown at real speed. Post-production editing of the screenshot was limited to spatial adjustments (cropping and repositioning) for improved visual presentation, with no modification to original content.

Supplementary Section 9: The denoising performance of different methods on calcium imaging data.

The calcium imaging data was originally sampled at 8 Hz. To facilitate comparison, the playback speed has been increased 11-fold.

Despite the heavy contamination of the raw data by noise, which distorts neuronal morphology and contours, the video illustrates the application of FAST alongside four other denoising methods. FAST effectively restores neuronal structures, revealing clear synaptic details and sharp cell boundaries, even in high-noise regions. In contrast, other methods often produce over-smoothed transitions that obscure fine structural details or introduce artifacts that compromise the fidelity of the restored images.

Supplementary Section 10: FAST enhances single-cell voltage imaging, with a comparison to electrophysiological signals.

The video features data originally sampled at 1,000 Hz, displayed at a video frame rate of 60 Hz. To facilitate observation and comparison, the playback speed has been slowed down by a factor of 32.

The raw data is severely contaminated by noise, which greatly deteriorates the spatial morphology and temporal trajectories of neurons. After applying FAST, cell boundaries become sharply defined, and the enhanced voltage waveforms show a significant correlation with electrophysiological recordings, demonstrating improved signal fidelity.

Supplementary Section 11: FAST enhances population voltage imaging of the zebrafish larval spinal cord.

The video features data originally sampled at 1,000 Hz and displayed at a frame rate of 60 Hz, with the playback speed slowed down by a factor of 10 to facilitate observation and comparison.

After denoising, neuronal structures previously obscured by noise become clearly visible. By manually delineating regions of interest (ROIs) for four neurons and extracting their voltage trajectories, we observe a significant reduction in noise while maintaining the authenticity of voltage transients.

Supplementary Section 12: FAST enhances two-photo dual-channel volumetric imaging of astrocytes.

Astrocyte dynamics were acquired at 1 Hz volumetric sampling rate. To facilitate comparison, the playback speed has been increased 15-fold.

In the video, the first channel displays morphological data, where FAST denoising significantly enhances the clarity of astrocyte structures, making previously indistinct features more

discernible. The second channel presents Ca^{2+} signals, where FAST effectively recovers the dynamic changes in calcium activity that were previously masked by severe noise, allowing for a more accurate observation of the underlying signal fluctuations.

Supplementary Section 13: Evaluating the impact of motion artifacts on FAST denoising performance.

To systematically evaluate the robustness of FAST to different degrees of motion artifacts, we generated simulated two-photon calcium imaging data using NAOMI and introduced mixed Poisson-Gaussian noise to mimic realistic imaging conditions. To simulate different motion levels, we introduced a random displacement between adjacent frames, with the magnitude (in pixels) varying across seven conditions. Seven motion conditions were tested: no motion (0), slow motion (0–1, 0–2, 0–3, 0–4, 0–5), and vigorous motion (4–5).

Each dataset was processed with FAST for denoising, and the structural similarity index (SSIM) was calculated by comparing the denoised images with the ground truth. As shown in Supplementary Figure 11, FAST achieved consistently high SSIM values under no motion and all slow motion conditions (SSIM = 0.59 for 0 and 0–1, 0.58 for 0–2 and 0–3, 0.57 for 0–4 and 0–5), indicating robust denoising performance despite minor motion. In contrast, the SSIM dropped substantially to 0.46 in the vigorous motion group (4–5), demonstrating that large inter-frame displacements can significantly impair denoising quality. For reference, the SSIM of the noisy raw images was 0.21 (dashed line in the figure). These results highlight the importance of motion stabilization for optimal denoising with FAST, especially under conditions of vigorous motion.

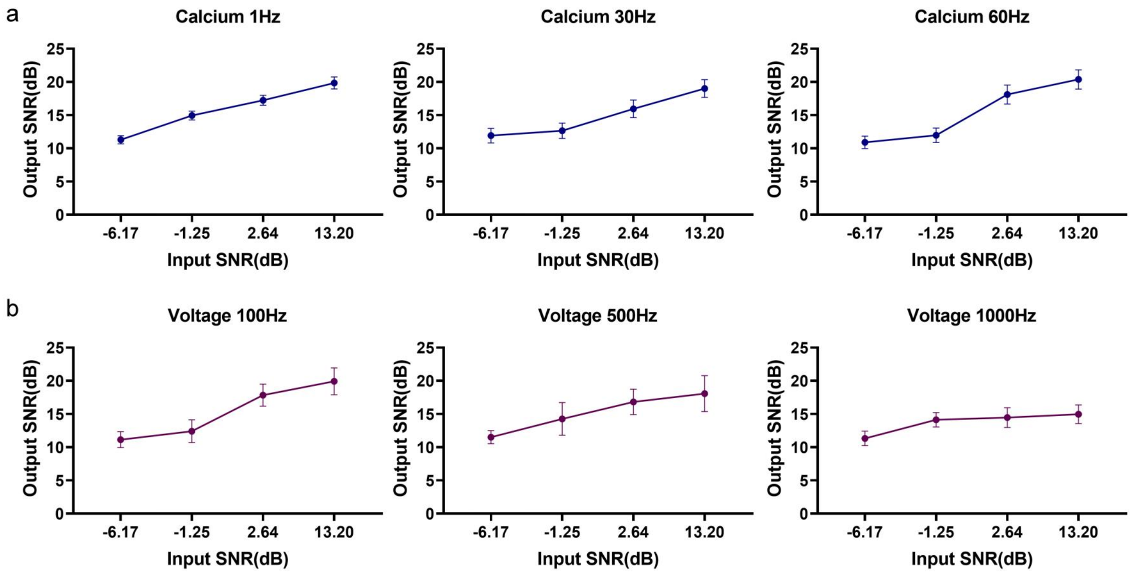

Supplementary Figure 1: Evaluating the performance of different SNR and imaging speeds. a, The denoising performance of FAST for calcium imaging under varying signal-to-noise ratios and imaging speeds. Noise-free 60Hz calcium imaging videos were generated using NAOMI, and validation data were obtained through frame subsampling and the addition of mixed Poisson-Gaussian noise. **b,** The denoising performance of FAST for voltage imaging under varying signal-to-noise ratios and imaging speeds. Noise-free 1,000Hz voltage imaging videos were generated using NAOMI, and validation data were obtained through frame subsampling and the addition of mixed Poisson-Gaussian noise. The line shows mean values and error bars represent the SD values.

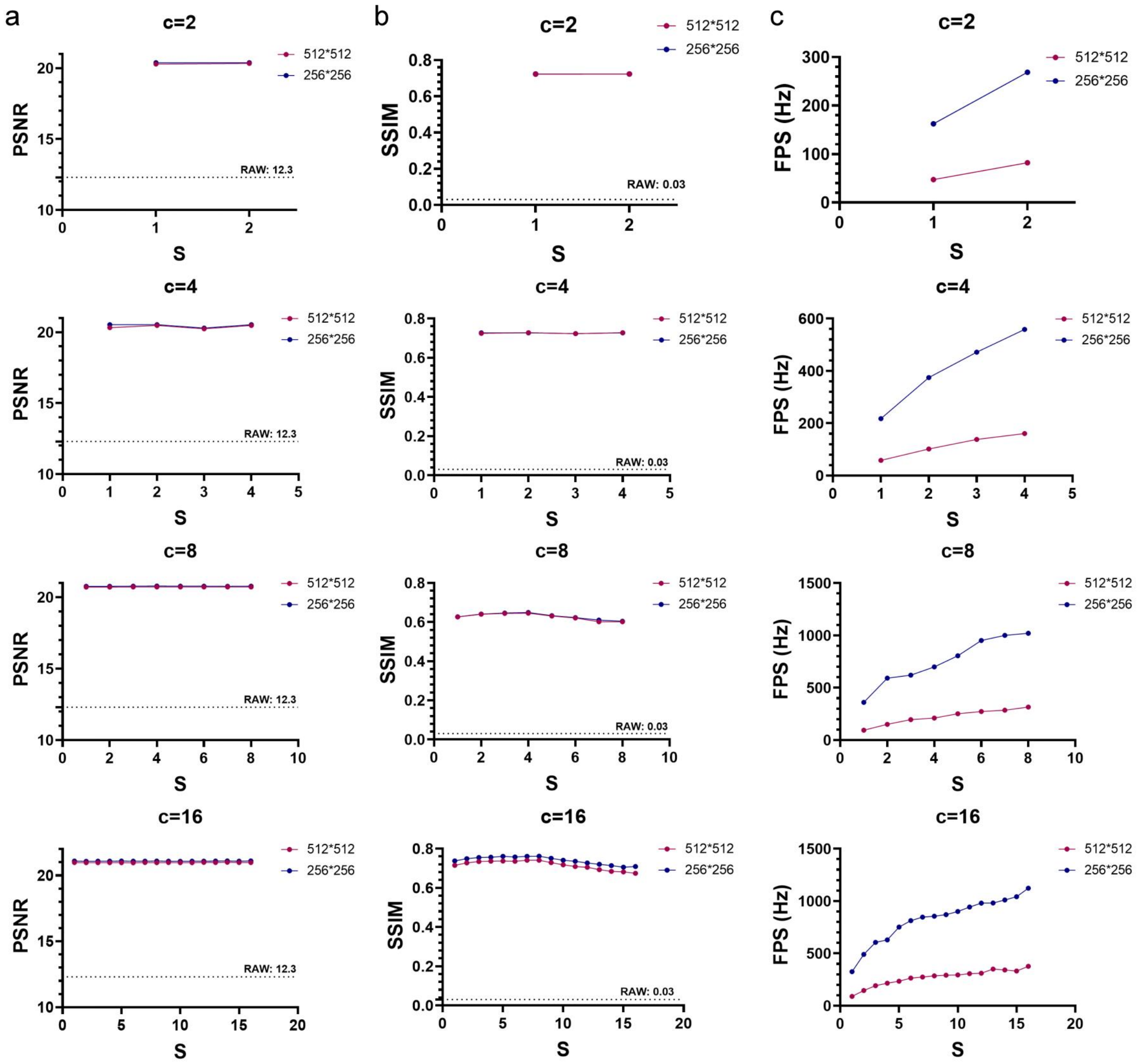

Supplementary Figure 2: Evaluation of hyperparameters. Examination of the sensitivity of the trade-off parameters, sliding window width C, and sliding window stride size S, using simulated data. a, The relationship between hyperparameters and denoising results in terms of PSNR. **b,** The relationship between hyperparameters and denoising results in terms of SSIM. **c,** The relationship between hyperparameters and processing speed in terms of FPS.

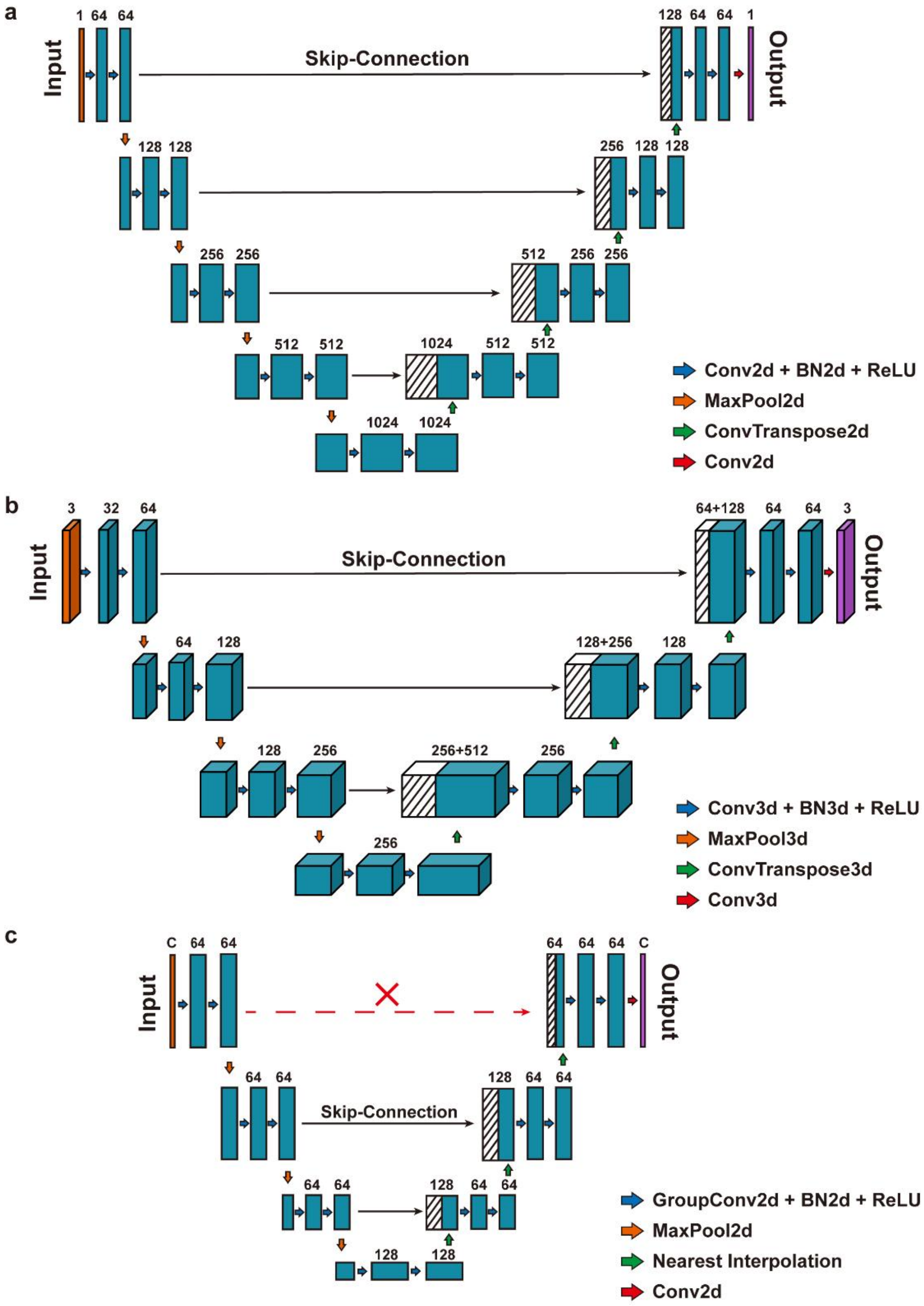

Supplementary Figure 3: Network architecture of FAST. a, The architecture of the classical 2D U-Net. The U-Net consists of an encoder (downsampling path) and a decoder (upsampling path), with skip connections linking corresponding layers to preserve spatial information. Typically, the U-Net architecture includes four downsampling layers in the encoder, with the number of feature map channels doubling at each layer, progressively capturing more abstract spatial features. **b**, The architecture of the 3D U-Net. This network extends the classical U-Net into the temporal domain, capturing spatiotemporal features through 3D convolutions. While this approach can improve the extraction of temporal dynamics, it introduces a significantly larger number of parameters and increases computational complexity, often demanding more memory

and processing power. **c**, The architecture of FAST, as proposed in this study. In contrast to both the classical and 3D U-Net architectures, our network maintains a constant number of feature map channels at 64 throughout both the encoder and decoder, avoiding the parameter-intensive operations of channel doubling and halving. All convolution operations are implemented as 2D Group Convolutions, with each group processing 2 channels. This design reduces the number of parameters and computational cost while enabling independent feature extraction within each group, which helps mitigate interference between groups and avoids artifacts caused by temporal signal aliasing. Additionally, the topmost skip connection has been removed, streamlining the network further. The number of input and output channels, denoted as, corresponds to the number of frames in the input image sequence, allowing FAST to efficiently process spatiotemporal information while avoiding the computational overhead typically associated with 3D convolutions.

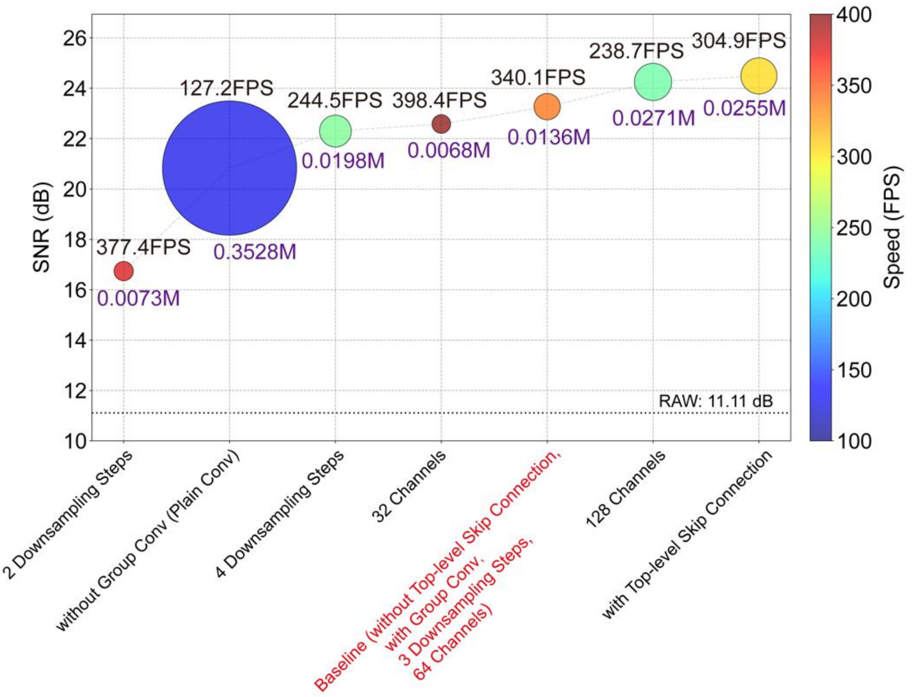

Supplementary Figure 4: Quantitative comparison of different network variants based on ablation experiments. The horizontal axis shows the network architecture variants, with the "Baseline (without Top-level Skip Connection, with Group Conv, 3 Downsampling Steps, 64 Channels)", which is the parameter setting chosen in our main manuscript, highlighted in red. The vertical axis represents the denoising performance measured by SNR (dB) for each model. The SNR of the raw input data (11.11 dB) is indicated by a horizontal dashed line for reference. Each point corresponds to a specific model variant; bubble size is proportional to the number of model parameters (in millions), and color represents processing speed (FPS), mapped using the jet colormap (blue indicates slower speed, red indicates faster speed). Variants include the baseline, a version without group convolutions (Plain Conv), a variant with an added top-level skip connection, models with 2 or 4 downsampling steps, and models using 32 or 128 channels. This visualization summarizes the trade-offs between model complexity, denoising performance, model size, and inference speed.

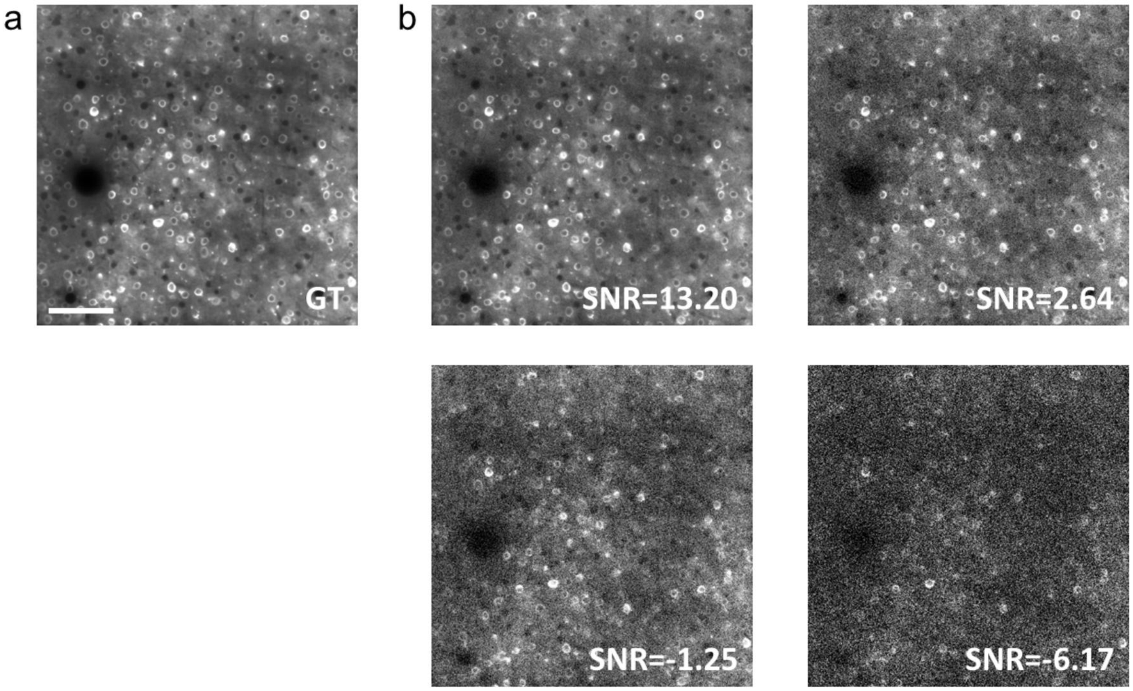

Supplementary Figure 5: *In silico* simulation of calcium imaging data. a, We utilize NAOMI to simulate noise-free two-photon calcium imaging videos as the ground truth. **b,** Mixed Poisson-Gaussian noise at varying levels is added to the ground truth. Scale bar, 100 μm.

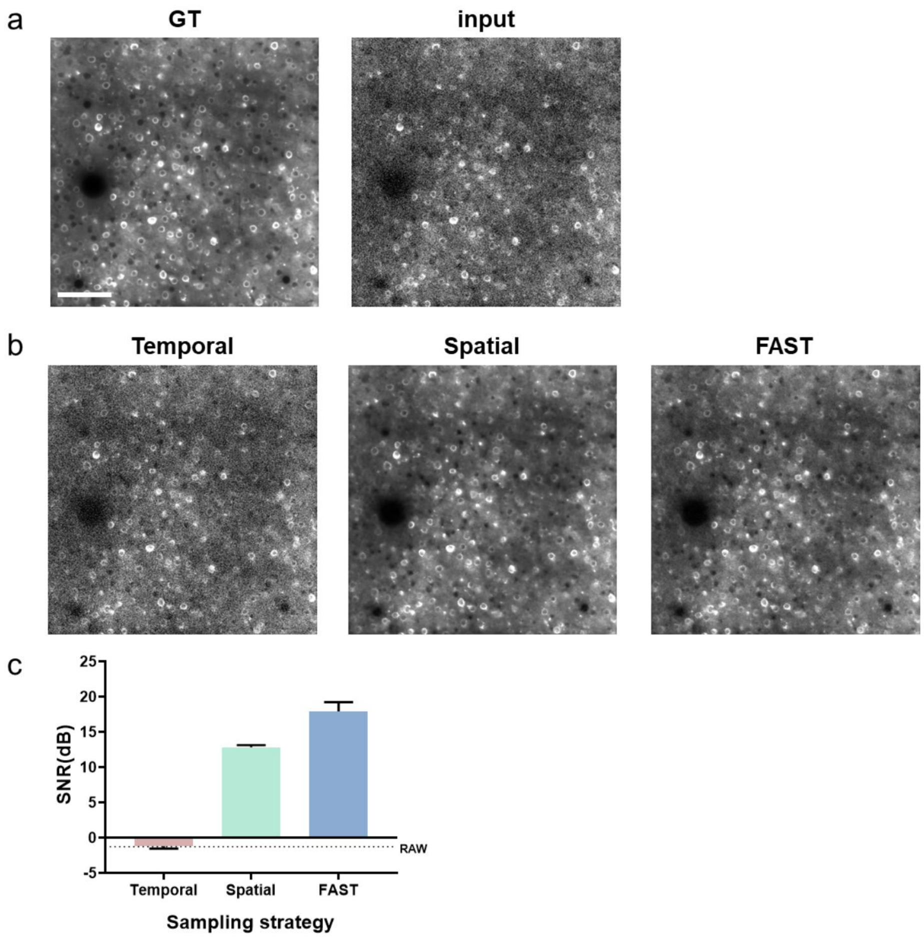

Supplementary Figure 6: Evaluating the sampling strategy of FAST. a, Noise-free simulated two-photon calcium imaging videos, to which mixed Poisson-Gaussian noise is added, serve as the input for the denoising network. **b,** Denoising results obtained from three sampling strategies: temporal sampling where input and target are temporally adjacent, spatial sampling where input and target are spatially adjacent, and FAST sampling which is spatiotemporally adjacent. **c,** The denoising performance of the three sampling strategies indicates that FAST outperforms both spatial and temporal sampling. Scale bar, 100 μm.

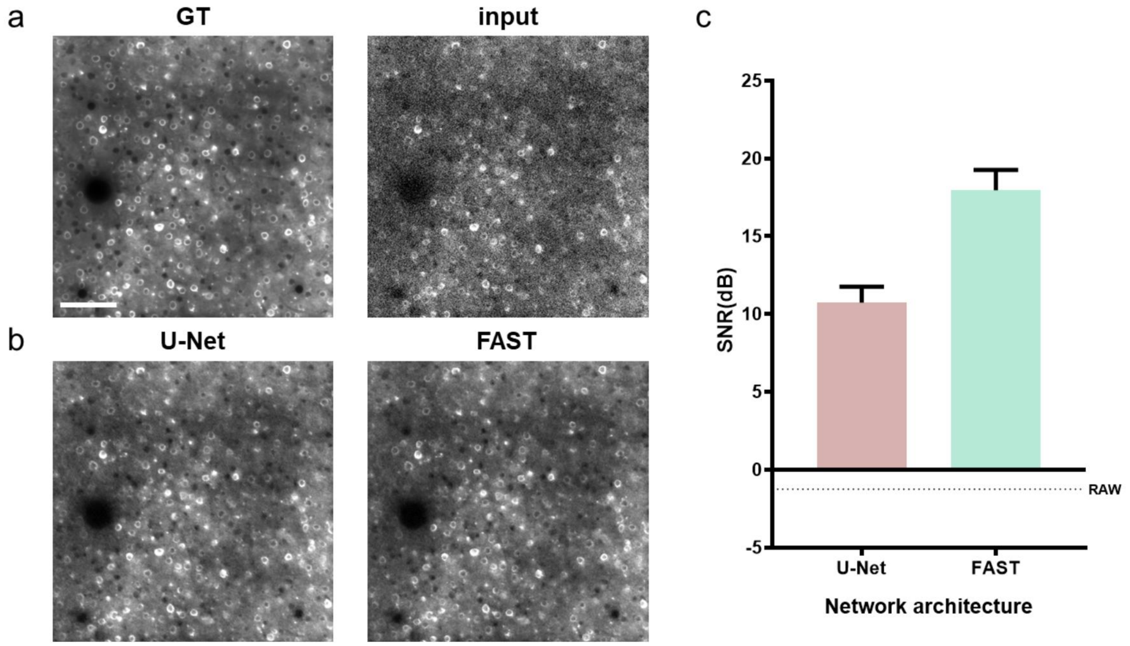

Supplementary Figure 7: Evaluating the network architecture of FAST. a, Noise-free simulated two-photon calcium imaging videos, which were augmented with mixed Poisson-Gaussian noise, were used as inputs for the denoising network. **b,** Denoising results are presented for both the classical U-Net network and the proposed FAST network, utilizing a spatiotemporal sampling strategy. **c,** The FAST network architecture demonstrates superior denoising performance compared to the classical U-Net network. Scale bar, 100 μm.

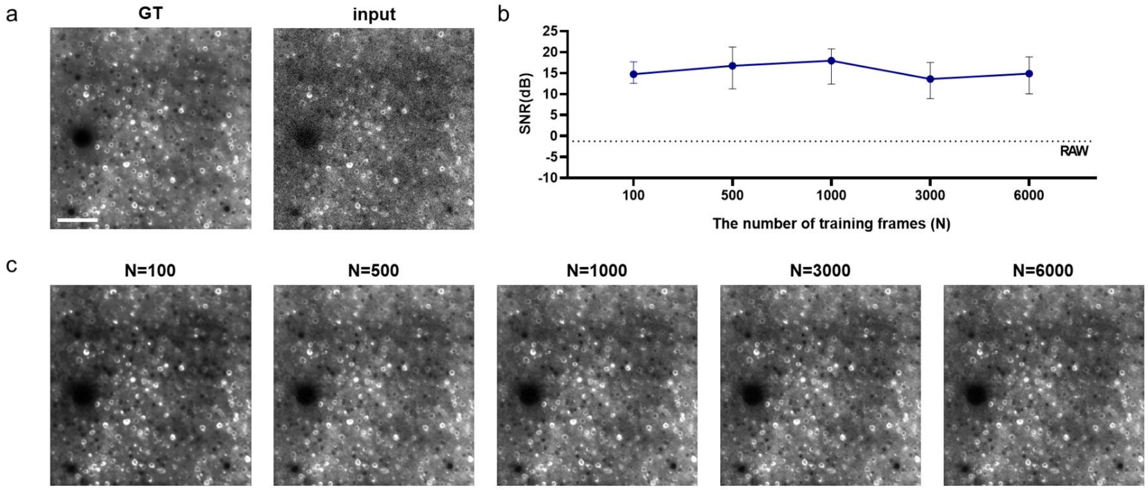

Supplementary Figure 8: Evaluating the data dependency of FAST. a, Noise-free simulated two-photon calcium imaging videos, to which mixed Poisson-Gaussian noise is added, serve as the input for the denoising network. **b,** Differences in denoising performance among models trained with varying numbers of frames. The line indicates mean values, and error bars represent the minimum and maximum values. **c,** Example images following denoising. Scale bar, 100 µm.

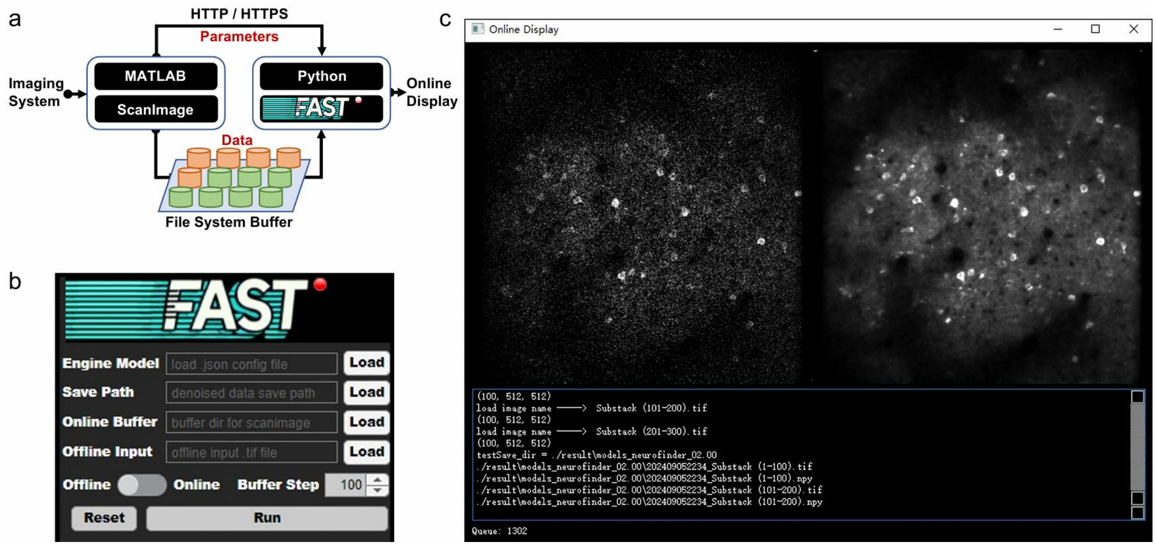

Supplementary Figure 9: FAST Graphical User Interface Overview. a, Image Acquisition and Processing Workflow. The FAST system employs ScanImage, a MATLAB-based software, for image acquisition. FAST integrates two main components: a MATLAB-based application and a Python-based deep learning denoising module. Communication between these components occurs via HTTP/HTTPS protocols for parameter exchange. Acquired data is buffered and subsequently accessed by the Python module for real-time processing and visualization. Post-processing, the denoised data is saved to the hard disk. **b,** MATLAB-Based Application Interface. The interface of the MATLAB-based FAST application is depicted. **c,** Python-Based Online Display Interface. The online display interface of the Python-based FAST module is shown.

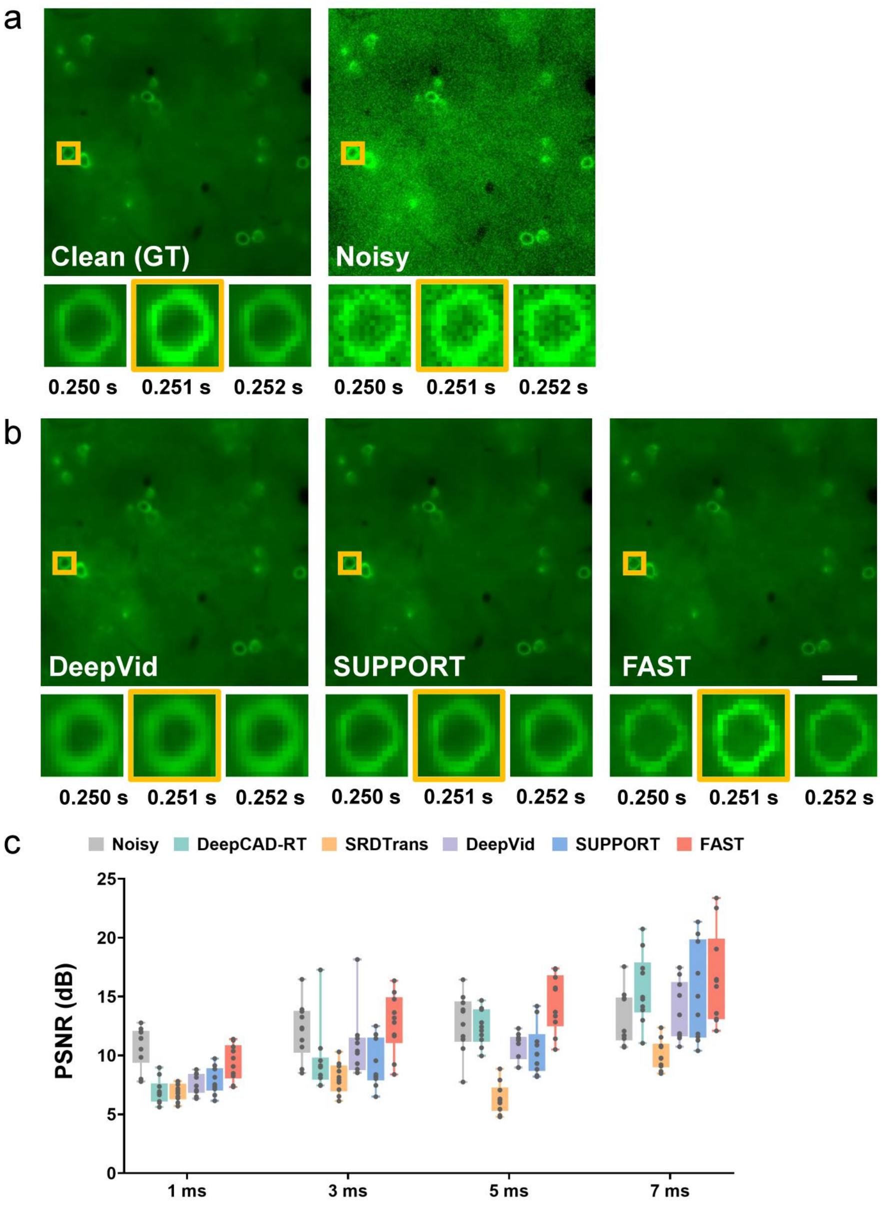

Supplementary Figure 10: Evaluation of FAST Performance in Fast Dynamics. a, Clean voltage imaging videos with 1 ms spike width were first generated to simulate fast dynamics, followed by the addition of mixed Poisson-Gaussian noise to replicate realistic imaging conditions. **b,** Denoising results from three methods applicable to voltage imaging are presented. A neuronal region of interest (ROI) was manually selected for demonstration, focusing on a 3 ms time window centered at the spike peak. **c,** Using the same approach, simulated datasets were generated with spike widths ranging from 1 ms to 7 ms. Voltage traces were extracted from manually selected neuronal ROIs, and the ΔF/F PSNR values were compared across noisy images, denoised results from five different methods, and the ground truth (GT). Scale bar, 50 µm.

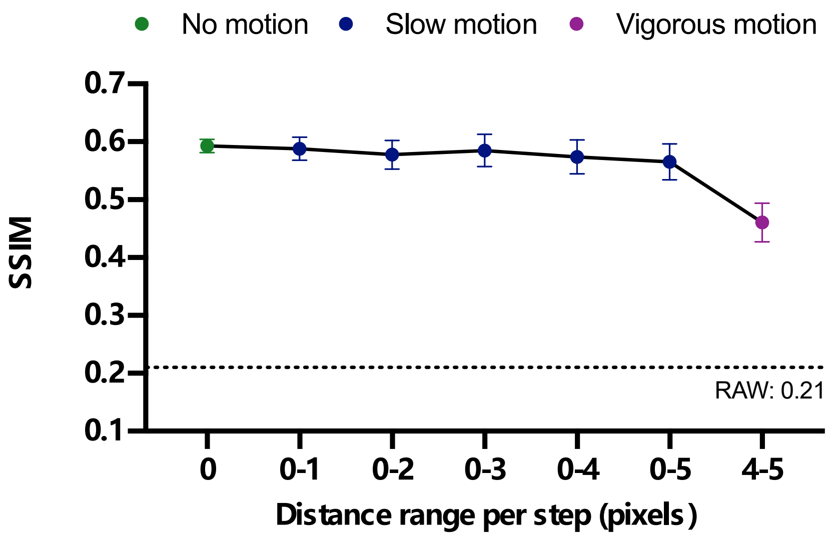

Supplementary Figure 11: Evaluating the impact of motion artifacts on FAST denoising performance. Simulated two-photon calcium imaging data were generated using NAOMI, with mixed Poisson-Gaussian noise artificially added. Seven groups of data were generated by introducing random rigid motion between adjacent frames with varying distance ranges per step (in pixels): no motion (0), slow motion (0–1, 0–2, 0–3, 0–4, 0–5), and vigorous motion (4–5). Each dataset was denoised using FAST, and the structural similarity index (SSIM) between the denoised results and the ground truth was calculated. The x-axis indicates the magnitude of simulated motion, defined as the range of random displacement (in pixels) introduced between adjacent frames for each condition. The y-axis shows the corresponding SSIM values (mean ± SD). The dashed line indicates the SSIM (0.21) of the noisy raw images. FAST maintained high SSIM values under no motion and slow motion conditions (0: 0.59, 0–1: 0.59, 0–2: 0.58, 0–3: 0.58, 0–4: 0.57, 0–5: 0.57), but showed a marked drop in performance under vigorous motion (4–5: 0.46).

Supplementary Table 1: List of datasets used in this study.

Region	Cell type	Recording rate (Hz)	Probe	Imaging modality	Reference
Mouse vS1 (Fig. 2a)	neurons	8	GCaMP6s	two-photon microscopy	Code Neurofinder (http://neurofinder.codeneuro.org)
Mouse cortex L2/3 (Fig. 3a)	pyramidal cells	1,000	QuasAr6a + patch clamp	epifluorescence microscopy	Eom, M. et al. Nat Methods 20, 1581–1592 (2023).
Zebrafish spinal cord (Fig. 3d)	excitatory (vGlut2a)	1,000	zArchon1	light sheet microscopy	Xie, Michael E. et al. Cell Reports 35.1 (2021).

Supplementary Table 2: List of denoising methods compared in this study.

Method	Repository URL	Framework	Network configuration	Reference
DeepCAD-RT	https://github.com/cabooster/DeepCAD-RT	PyTorch	Changed patch_xy to 50, patch_t to 25 from default	Li, X. et al. Nat Biotechnol 41, 282–292 (2023).
SRDTrans	https://github.com/cabooster/SRDTrans	PyTorch	Changed patch_x to 32, patch_t to 32 from default	Li, X. et al. Nat Comput Sci 3, 1067–1080 (2023).
DeepVid	https://github.com/bu-cisl/DeepVID	TensorFlow	Default	Platisa, J. et al. Nat Methods 20, 1095–1103 (2023).
SUPPORT	https://github.com/NICALab/SUPPORT	PyTorch	Default	Eom, M. et al. Nat Methods 20, 1581–1592 (2023).

Reference:

1. Song, A., Gauthier, J. L., Pillow, J. W., Tank, D. W. & Charles, A. S. Neural anatomy and optical microscopy (NAOMi) simulation for evaluating calcium imaging methods. *J. Neurosci. Methods* **358**, 109173 (2021).
