# Supplementary material for: Real-time self-supervised denoising for high-speed fluorescence neural imaging
Source: Nat Commun. 2025 Oct 24;16:9396. doi: 10.1038/s41467-025-64681-8 (PMC12552699; doi:10.1038/s41467-025-64681-8)
Supplement: Supplementary file 2 — Description of Additional Supplementary Files [file 41467_2025_64681_MOESM2_ESM.pdf]

Description of Additional Supplementary Files

Supplementary Video 1: Real-time denoising of two-photon calcium imaging in mice with FAST. We used Matlab-based ScanImage software for image acquisition. Before starting the experiment, imaging control parameters were preset. FAST was then launched, and the data read and save paths, model path, and buffer size were configured. The FAST system consists of two main components: a Matlab-based application and a Python-based deep learning denoising module. Acquired image data is buffered and accessed in real time by the Python module for denoising and visualization. During the experiment, both the raw and denoised images are displayed simultaneously, enabling real-time comparison. After processing, the denoised data is automatically saved to disk. The video demonstrates an acquisition rate of 30 Hz at 512×512pixels and is shown at real speed. Post-production editing of the screenshot was limited to spatial adjustments (cropping and repositioning) for improved visual presentation, with no modification to original content.

Supplementary Video 2: The denoising performance of different methods on calcium imaging data. The calcium imaging data was originally sampled at 8 Hz. To facilitate comparison, the playback speed has been increased 11-fold. Despite the heavy contamination of the raw data by noise, which distorts neuronal morphology and contours, the video illustrates the application of FAST alongside four other denoising methods. FAST effectively restores neuronal structures, revealing clear synaptic details and sharp cell boundaries, even in high-noise regions. In contrast, other methods often produce over-smoothed transitions that obscure fine structural details or introduce artifacts that compromise the fidelity of the restored images.

Supplementary Video 3: FAST enhances single-cell voltage imaging, with a comparison to electrophysiological signals. The video features data originally sampled at 1,000 Hz, displayed at a video frame rate of 60 Hz. To facilitate observation and comparison, the playback speed has been slowed down by a factor of 32. The raw data is severely contaminated by noise, which greatly deteriorates the spatial morphology and temporal trajectories of neurons. After applying FAST, cell boundaries become sharply defined, and the enhanced voltage waveforms show a significant correlation with electrophysiological recordings, demonstrating improved signal fidelity.

Supplementary Video 4: FAST enhances population voltage imaging of the zebrafish larval spinal cord. The video features data originally sampled at 1,000 Hz and displayed at a frame rate of 60 Hz, with the playback speed slowed down by a factor of 10 to facilitate observation and comparison. After denoising, neuronal structures previously obscured by noise become clearly visible. By manually delineating regions of interest (ROIs) for four neurons and extracting their voltage trajectories, we observe a significant reduction in noise while maintaining the authenticity of voltage transients.

Supplementary Video 5: FAST enhances two-photo dual-channel volumetric imaging of astrocytes. Astrocyte dynamics were acquired at 1 Hz volumetric sampling rate. To facilitate comparison, the playback speed has been increased 15-fold. In the video, the first channel displays morphological data, where FAST denoising significantly enhances the clarity of astrocyte structures, making previously indistinct features more discernible. The second channel presents Ca2+ signals, where FAST effectively recovers the dynamic changes in calcium activity that were previously masked by severe noise, allowing for a more accurate observation of the underlying signal fluctuations.
